# Integrating Data Visualizations Into Digital Mental Health Care for Adults With Anxiety and Depression: Participatory Design and Case Study

**DOI:** 10.2196/90255

**Published:** 2026-04-24

**Authors:** Katherine Lim, Rebekah Bodner, Kathryn Taylor Ledley, Shiwei Wang, Luke S Scheuer, John Torous

**Affiliations:** 1 Division of Digital Psychiatry, Beth Israel Deaconess Medical Center, 330 Brookline Ave, Boston, MA, 02215, United States, 1 6176676700; 2 Boston University, Boston, MA, United States

**Keywords:** data visualization, digital phenotyping, patient engagement, mental health, digital mental health, digital navigator

## Abstract

**Background:**

Digital phenotyping offers unprecedented opportunities for capturing real-time mental health data through smartphones, yet translating this data into clinically actionable insights remains challenging. While smartphones can generate nearly one million data points per patient per day, health care systems have struggled to incorporate even basic ecological momentary assessment data into routine care workflows.

**Objective:**

This paper presents a model for clinician-facing data visualizations that can be shared with patients to increase understanding of mental health symptoms and enhance shared decision-making. We describe (1) a participatory design process through which visualizations were cocreated with clinicians; (2) integration of these visualizations into a Digital Navigator-supported (DN) workflow; and (3) a case example illustrating how data visualizations can enhance patient insight and support treatment adjustments.

**Methods:**

This work was conducted within the Digital Clinic program at Beth Israel Deaconess Medical Center. Fifteen clinicians and 3 clinical supervisors participated in a participatory design process to develop visualizations meeting clinical workflow needs. Data visualizations were integrated into weekly DN sessions following a 3-phase model (guide, refinement, and autonomy) based on self-determination theory.

**Results:**

Six visualization types were developed: gauge charts for engagement behaviors, symptom trajectory graphs, correlation matrices linking passive and active data, sleep visualizations, polar/radar charts for multidimensional assessment, and passive-active data integration graphs. A clinical case demonstrates how these visualizations, when delivered through structured DN facilitation, supported patient engagement, behavioral insight, and autonomous self-management across an 8-week treatment program.

**Conclusions:**

Thoughtfully designed data visualizations, when developed collaboratively with clinicians and delivered through structured support, can transform digital phenotyping from a technical capability into a practical tool for enhancing engagement, promoting behavioral insight, and supporting self-management in digital mental health care. Future research should examine how this approach affects therapeutic alliance and clinical outcomes across diverse patient populations.

## Introduction

Patient engagement is essential to delivering effective health care. Despite many digital health tools offering impressive efficacy data, their real-world effectiveness has been less impressive due to low real-world engagement [1]. While there are many pathways to improve engagement, extensive efforts in better design, patient cocreation, and gamification have yielded only modest improvements [2]. Another pathway, data sharing, has been less explored. In this paper, we examine how data visualization can drive clinical engagement with digital health tools.

Data sharing in the era of digital health holds untapped potential. The widespread availability of smartphones, owned by over 90% of Americans and 50% of the global population, has created unprecedented opportunities for capturing real-time health data [3,4]. Through digital phenotyping, the process of quantifying human behavior using personal smartphone data, clinicians can access rich information, including sleep patterns, physical activity, mobility routines, screen time, and self-reported symptoms through ecological momentary assessments (EMAs). Numerous reviews highlight that such data can inform preventive, predictive, and more responsive care across the spectrum of mental illnesses [5,6].

However, this data richness presents a formidable barrier to clinical integration and shared decision-making. Modern smartphones can generate nearly one million data points per patient per day when continuously sampling GPS and accelerometer data at standard frequencies [7]. Health care systems have already struggled to incorporate even basic EMA survey data into routine care workflows, and the volume and complexity of passive sensor data amplify these challenges [8]. Bringmann et al [8] developed ESMvis, one of the few tools created to display individual experience sampling method (ESM) data for clinical use. Their work highlights both the promise and the challenge of turning detailed, real-time data into formats that clinicians can quickly understand during short appointments [8]. More broadly, research shows several barriers to using ESM in routine care: limited clinician time, uncertainty about how to interpret fluctuating moment-to-moment data, and a lack of clear workflows for reviewing results [9-11]. Qualitative studies also find that while patients appreciate the self-reflection encouraged by ESM, they often want structured guidance to help them understand their data [11]. Likewise, clinicians report preferring clear, actionable summaries rather than raw data streams. Emerging evidence suggests that both clinicians and patients are receptive to digital phenotyping data when supported by clear, interpretable formats. A 2022 survey of UK clinicians found openness to incorporating digital phenotyping data when interpretation support was available [12], and related research indicates clinicians want actionable information summarized from raw behavioral data rather than unprocessed outputs [13]. Similarly, patients express willingness to share personal data if they trust the recipients and can derive meaningful insights from what they share [14].

To address this challenge, our team at the Digital Clinic at Beth Israel Deaconess Medical Center (BIDMC) has developed data visualizations that translate complex digital phenotyping data into clinically interpretable formats, supported by a structured workflow for their use in care [15,16]. Central to our model is the Digital Navigator (DN), a trained member of the care team who supports technology-enabled care and guides patients through the interpretation of visual data reports in structured weekly sessions [17]. Unlike models where patients access data independently, this approach ensures that visualizations function as communication tools that support collaborative decision-making rather than standalone interventions. This workflow positions the DN as a guide who helps patients progress toward autonomy in understanding their own data.

This structure mirrors the broader Digital Clinic model, in which digital tools, such as the mindLAMP smartphone application and weekly data reports, support reflection, shared understanding, and care coordination, while clinical treatment is delivered through clinician-led psychotherapy and skills-based interventions [18].

In this paper, we present a model for clinician-facing visualizations that can be shared with patients and targeted to their specific conditions to increase both patient and provider understanding of mental health symptoms and enhance shared decision-making around disease management. We illustrate (1) a participatory design process through which visualizations were cocreated with clinicians to meet clinical workflow needs, (2) integration of these visualizations into a DN-supported workflow, and (3) a detailed case example illustrating how data visualizations, when paired with structured facilitation, can enhance patient insight, contextualize symptom fluctuations, and support real-time treatment adjustments.

The visualizations depicted here are currently in use in both research and clinical settings within the Digital Clinic program [18,19]. The aim of this paper is to describe a replicable model for integrating clinician-informed data visualizations into a DN-supported clinical workflow for digital mental health care. Specifically, we aim to (1) document the participatory design process through which visualizations were developed, (2) describe how these visualizations are embedded within structured DN sessions, and (3) illustrate through a detailed clinical case example how this approach can enhance patient engagement, promote behavioral insight, and support the development of autonomous self-management skills.

## Methods

### Ethical Considerations

This work was conducted as a quality improvement initiative within the Digital Psychiatry Clinic program at BIDMC. The project was reviewed by the BIDMC Institutional Review Board and determined to be a quality improvement activity, not human subjects research, and therefore exempt from Institutional Review Board oversight (Protocol # 2022D000016). The case example presented was deidentified, and identifying details have been modified to protect patient privacy. No compensation was provided as part of this work.

All data visualizations were generated using Python-based tools applied to data stored within institutional infrastructure, with processing supported by Cortex, an open-source pipeline developed by the Division of Digital Psychiatry specifically for mindLAMP-based studies. Phenotyping data include passive metrics (such as entropy, home duration, screen duration, and step count) and self-reported survey responses collected at daily and weekly frequencies. Passive signals are aggregated and deidentified so that precise locations and raw GPS traces are not stored or displayed to clinicians. In the current implementation, all identifiable clinical and digital phenotyping data remain under the control of BIDMC and are not shared with external third parties for commercial use.

### Participants and Clinical Context

This work was conducted within the Digital Clinic program at the Division of Digital Psychiatry at BIDMC, an affiliated teaching hospital of Harvard Medical School. The Digital Clinic provides an 8-week, evidence-based virtual treatment program for adults primarily diagnosed with mild to moderate anxiety or depression [20]. The program consists of (1) the mindLAMP smartphone application, which collects EMAs, passive behavioral data, and sensor-derived activity metrics; (2) weekly clinician sessions focused on psychotherapy or skills-based interventions; and (3) weekly meetings with a DN, who supports patient engagement with the digital tools and assists with interpretation of the visualized data.

**Figure 1.**
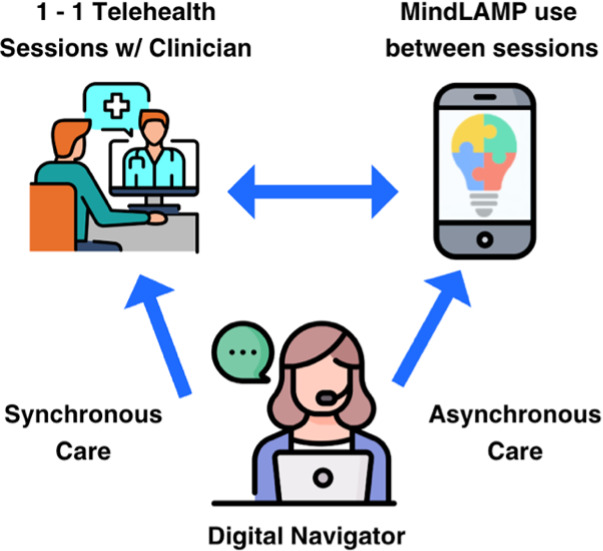
Digital clinic care model workflow.

Patients complete the Patient Health Questionnaire-9 (PHQ-9) and Generalized Anxiety Disorder-7 (GAD-7) on a weekly basis through the mindLAMP application. In addition, patients respond to brief daily EMA surveys administered once per day via push notification, comprising 3 single-item Likert-scale ratings that assess daily anxiety, daily mood, and daily difficulty functioning. Passive data collection occurs continuously through the smartphone’s built-in sensors, including GPS (from which entropy, hometime, and distance metrics are derived), accelerometer (used to estimate step count and sleep), and screen state (used to estimate screen time and, in combination with accelerometer data, sleep duration) [21]. At intake, midpoint (wk 4), and completion (wk 8), patients also complete a broader assessment battery that includes measures of emotional self-awareness, perceived social support, self-efficacy, motivation, and digital literacy. Figure 1 shows a schematic of the digital clinic team and tools.

### The Digital Navigator Role and Implementation

DNs are research assistants or clinical research coordinators with at least a bachelor’s degree who serve as technology and engagement support members of the care team. Training includes learning the mindLAMP app and its reports, practicing motivational interviewing, and completing supervised practice sessions [22]. Weekly meetings with clinicians and supervisors support case discussion and skill development. DNs are also trained to recognize and escalate situations requiring clinical attention, such as worsening symptoms or safety concerns.

DNs work alongside but separately from clinician-led therapy, meeting patients for weekly 30-minute virtual sessions to review data reports, resolve technical issues, and support data comprehension. After each session, they document key observations and data patterns in a shared note for the treating clinician, enabling efficient information transfer without additional data review time. DNs do not provide psychotherapy or make clinical decisions; their role is to support data engagement and clinical communication. This aligns with the broader framework by Wisniewski and Torous, which presents DNs as a scalable role adaptable to various professional backgrounds [22].

For conflict resolution and clinical oversight, weekly case reviews with supervising clinicians address ambiguous data patterns or engagement concerns. When disagreement or uncertainty arises, DNs follow the supervising clinician’s recommendations regarding safety planning or treatment adjustments. Discrepancies between patient statements and DN inferences are clarified during sessions, with the patient’s interpretation documented in their chart.

DNs also act as advocates for patient preferences. If a patient feels overwhelmed by certain data streams, the DN offers to pause specific data collection while continuing therapy and communicates this to the clinician. Individual visualization components (eg, sleep or mobility graphs) can be removed from the report to align with patient comfort while preserving clinical usefulness.

The mindLAMP application includes free-text journaling for patients to record reflections, and relevant excerpts can be incorporated into weekly reports. During sessions, visualization interpretations (eg, “my anxiety tends to increase on d with high screen time”) are developed collaboratively and documented as the patient’s own statements. This supports the coproduction of meaning: visual patterns are presented, jointly interpreted, and recorded, reflecting the patient’s perspective. When disagreements arise, DNs prioritize the patient’s account and adjust their documentation accordingly.

Lack of engagement is operationalized as 0% completion of daily surveys or exercises over the preceding week. The DN explores potential barriers (eg, difficulty locating surveys, forgotten assignments, reduced motivation) and collaboratively proposes solutions. If a patient misses both DN and clinician sessions in one week, the DN emails to inquire and offer rescheduling; clinicians are alerted after every missed appointment. Currently, the program does not use automated notifications or messaging for nonadherence; all outreach and monitoring are conducted manually by DNs and clinicians. DNs review weekly PHQ-9 and GAD-7 scores and typically notify the clinician when an increase of approximately 3 or more points is observed. All changes in symptom scores are documented in the patient’s note, but larger increases prompt same-day communication from the DN to the clinician so that potential treatment adjustments can be considered at the next scheduled visit.

### Visualization Needs Assessment and Design

To ensure visualizations would meet clinical workflow needs, 15 clinicians (bachelor's degree or higher) and 3 clinical supervisors (PhD-level) from the Digital Clinic participated in a participatory design process. This work built off of prior published papers [21,22] that established foundational principles for patient-centered digital health tools. All participants routinely review mindLAMP-generated reports and integrate digital phenotyping insights into therapeutic work.

The process consisted of 4 iterative cycles over approximately 3 months. Each cycle included a structured group feedback session reviewing draft visualizations for clinical relevance, interpretability, and workflow burden. Participants provided feedback on clarity, usefulness for DN and clinician-patient discussions, and data quality concerns. Decisions were reached through consensus, with disagreements resolved by prioritizing the majority clinical perspective while documenting alternatives. Between cycles, the team refined prototypes based on the preceding feedback.

Through structured discussions, clinicians identified priority visualization needs:

1. Engagement behaviors (survey completion patterns and exercise adherence)
2. Symptom-severity trajectories for depression, anxiety, and difficulty functioning at weekly and daily levels
3. Correlation matrices linking passive digital phenotyping features and EMA responses
4. Sleep duration and patterns
5. Polar charts displaying intake, interim, and completion survey scores

The design team applied three core principles: (1) concept-oriented presentation highlighting key patterns, (2) simplicity and accuracy supporting varying data literacy levels, and (3) contextual annotations and consistent color schemes clarifying meaning and flagging concerning changes. These priorities aligned with DN workflows, where DNs orient patients to weekly reports, explain symptom trends, and contextualize behavioral patterns.

Key refinements across development cycles included several usability and clarity improvements. First, color-coded indicators for data quality (red=low completeness; green/blue=sufficient) were added after clinicians reported difficulty distinguishing low-quality data from meaningful signals. Second, axis labels were simplified and paired with brief contextual summaries in response to feedback that the original labels were overly technical. Third, meaningful changes were visually highlighted to aid treatment decisions, addressing supervisors’ emphasis on quickly identifying clinically significant shifts. Many patients found correlation matrices difficult to interpret without guidance, leading to the addition of contextual annotations and a shift to introducing these visualizations during DN sessions rather than as independent, patient-facing materials. Clinicians ultimately reached consensus on a final set of 6 visualization types, confirming they could be feasibly reviewed within the time limits of clinical and DN sessions. These refinements supported full integration of DNs into the Digital Clinic. Data collection, processing pipelines, and technical implementation details are described in Multimedia Appendix 1 [23,24] and Multimedia Appendix 2, and we share all code to create these visualizations to ensure that the work is generalizable to the community [23].

In addition to clinician-focused participatory design, the visualization reports and DN workflow were iteratively refined based on patient feedback obtained during the 8-week program. At the final session, DNs conduct a qualitative interview reviewing the final data report with the patient, ask about the usability and relevance of the visualizations, and document suggestions for improvement that inform subsequent iterations of both the report and session structure. Patients valued seeing engagement patterns early but found the original layout overwhelming. Accordingly, the report was restructured to lead with gauge charts before more complex visualizations. Patients also requested clearer labeling of normal variability versus clinically meaningful change, leading to shaded threshold bands and plain-language annotations. Additional refinements included contextual annotations for irregular schedules (eg, shift work or academic deadlines) in sleep visualizations and a more accessible color palette with textual labels after some patients reported difficulty distinguishing colors. Together, these patient-driven adjustments illustrate how user feedback within routine care directly shaped both the content and presentation of the visualization reports.

### Overview of Key Visualization Components in the Data Report

**Figure 2.**
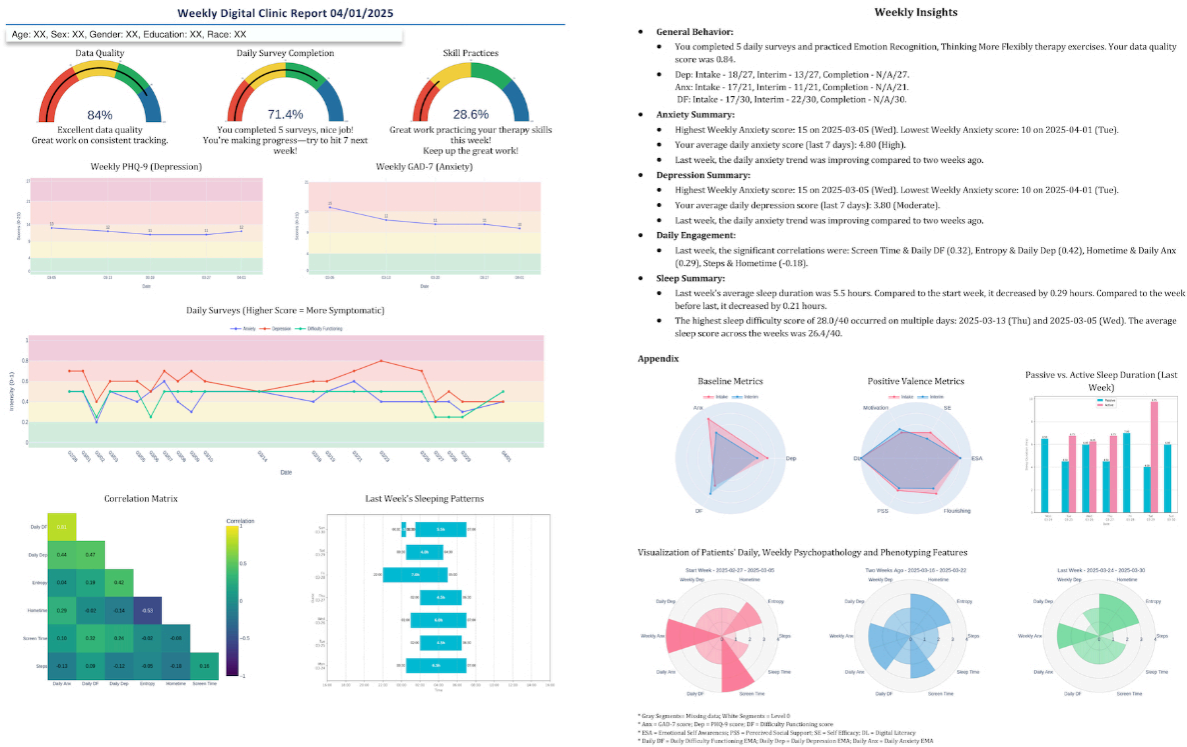
Complete data report sample showing the core 2 pages, which can be delivered digitally or printed.

The weekly data report integrates multiple visualization types, each serving distinct clinical functions, and is provided at every clinical or research visit. While the specific details may vary across studies or sessions depending on the data collected and the needs identified by patients, clinicians, or researchers, the basic structure remains consistent, as shown in Figure 2.

Table 1 provides more details on each visual type.

Each visualization type was designed to support both clinical decision-making and patient insight-building. The integration of multiple formats within a single weekly report enables a comprehensive understanding of symptom patterns, behavioral correlations, and treatment progress while accommodating diverse learning styles and levels of data literacy. DNs use these visualizations flexibly, emphasizing different components based on individual patient needs, clinical concerns, and phase of treatment.

**Table 1.** Overview of visualization components in weekly data reports.

Visualization type	Visual format and key features	Data displayed	Clinical function
Gauge charts	Color-coded gauge charts using traffic-light system (red/yellow/green-blue)	Data quality, survey completion rates, and skills practice adherence	Quickly assess engagement patterns and identify weeks requiring additional support
Symptom trajectory graphs	Line charts with clinical thresholds marked; separate weekly and daily views	PHQ-9^a^ (depression) and GAD-7^b^ (anxiety) scores over time	Track symptom trends, identify treatment response patterns, and distinguish persistent symptoms from transient mood fluctuations
Correlation matrices	Heat maps with color-coded correlation strength (warm colors=positive correlations, cool colors=negative correlations; intensity=strength)	Spearman correlations between passive behavioral metrics (GPS-derived entropy, step count, hometime, screen time, and sleep duration) and active self-report measures (daily anxiety, depression, and difficulty functioning)	Identify behavioral targets for intervention by revealing which routines most strongly associate with symptom patterns
Sleep visualization	Column charts with contextual annotations for sleep patterns	Daily sleep duration with visual markers	Make sleep patterns immediately visible and support discussions about sleep hygiene’s role in symptom management
Polar/radar charts	Multidimensional radar format with overlaid time points (intake-red, interim-blue, and completion-green)	Symptom domains (depression, anxiety, and difficulty functioning) and positive metrics (emotional self-awareness, perceived social support, self-efficacy, motivation, and digital literacy)	Enable rapid visual comparison across domains and time points to recognize multidimensional change patterns beyond single symptom reduction
Passive-active data integration graphs	Dual-axis line graphs overlaying behavioral metrics and symptom reports on shared timeline	Passive behavioral data (step count, screen time, and entropy) overlaid with active symptom reports (daily anxiety and depression) showing temporal alignment	Reveal relationships between behaviors and symptoms to support prediction making about behavioral-symptom connections

a PHQ-9: Patient Health Questionnaire-9.

b GAD-7: Generalized Anxiety Disorder-7.

### Integration of Visualizations Into Digital Navigator Workflow

#### Overview

**Figure 3.**
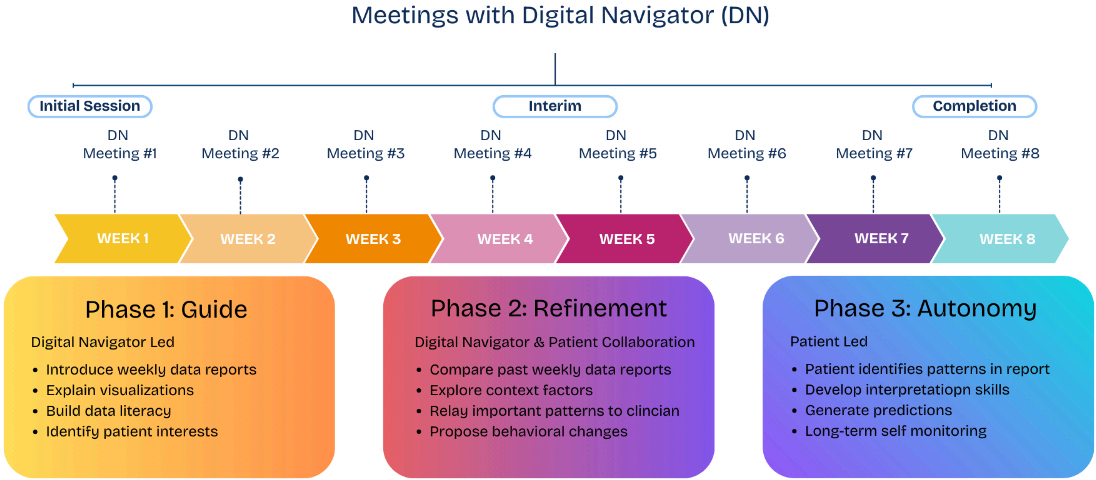
Digital navigator phases across an 8-week timeline.

DN sessions serve as the primary means for patient-facing interpretation of visual data reports. The DN workflow follows a 3-phase model based on the DOORS (Digital Outreach for Obtaining Resources and Skills) framework self-determination theory: guide, refinement, and autonomy [25]. Figure 3 illustrates how these phases map onto the 8-week treatment timeline, showing the progression from DN-led sessions toward patient-led data interpretation.

#### Phase 1: Guide

During the first data-focused DN session, the DN introduces the patient to the weekly report, explaining how each visualization is generated, simplifying visual elements, helping interpret early patterns relative to daily routines, and identifying initial correlations. This establishes foundational data literacy for the subsequent sessions and builds initial confidence in engaging with personal health data. The DN also prioritizes the patient’s participation and self-interest by thoroughly going over the whole report to gauge which graph or visualization the patient found most enlightening.

#### Phase 2: Refinement

In weekly follow-up meetings, DNs and patients compare updated reports to prior weeks, examining changes in symptoms, behaviors, or data quality alongside potential contextual explanations (eg, work schedule shifts and sleep disruption). During this phase, DNs support the development of competency and skills in data interpretation. DNs relay notable patterns to clinicians, supporting treatment plan updates. Visualizations function as prompts for collaborative problem-solving between the care team and patient, helping patients develop confidence in their ability to identify meaningful patterns and connect behavioral data to lived experience.

#### Phase 3: Autonomy

As patients develop their data literacy skills, DNs progressively shift toward supporting patient autonomy in data interpretation and self-management. Patients increasingly take the lead in identifying patterns, generating predictions about behavioral-symptom relationships, and proposing behavioral changes. This transition supports the development of long-term self-monitoring skills that may extend beyond the program.

At the final meeting, DNs and patients review overall trends across the 8-week program and gather structured feedback on the data visualization’s clarity, relevance, and how well visual patterns reflected their lived experiences.

## Results

### Overview

To illustrate how visualization design and DN workflow function in practice, we present a clinical case from a patient referred to the Digital Clinic with primary concerns related to anxiety. The patient is a current advanced degree student in their late 20s who was referred to the clinic regarding anxiety. This example demonstrates how weekly visual reports, passive-active data integration, and DN-guided interpretation supported insight and engagement across an 8-week treatment program.

### The Role of Visualizations for Collaborative Interpretation

Across the program, the DN played a central role in translating the data visualizations into actionable clinical insights. Through structured weekly meetings, the DN helped the patient build data literacy, validate visual patterns against lived experience, and develop a nuanced understanding of relationships between behavioral routines and mental health symptoms. The visualizations served as anchors for collaborative interpretation: the DN used symptom trajectories to track progress, correlation matrices to identify behavioral targets, and polar charts to illustrate multidimensional change over time.

The weekly data reports reviewed by the DN and the patient contained multiple visualization types working in concert. Each visualization format served as a distinct anchor point for different aspects of clinical understanding, and their integration created a comprehensive system for tracking, understanding, and responding to the patient’s mental health journey. Below, we describe how 3 core visualization types function as anchors across the guide, refinement, and autonomy phases of treatment.

### Tracking Progress and Contextualizing Change

Line graphs displaying weekly PHQ-9 (depression) and GAD-7 (anxiety) scores over time served as the primary anchors for monitoring treatment response and distinguishing between persistent patterns and transient fluctuations. These temporal visualizations provided both the patient and DN with a shared reference point for discussing symptom evolution.

### Early Validation of Visual Symptom Trajectories

**Figure 4.**
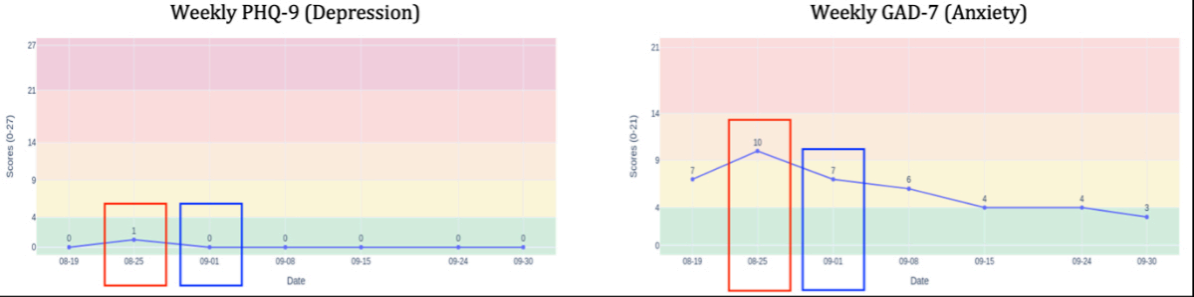
Weekly symptom trajectory graphs. GAD-7: Generalized Anxiety Disorder-7; PHQ-9: Patient Health Questionnaire-9.

During the initial session, the DN explained how the line charts were generated from weekly survey responses and pointed out clinical threshold lines that contextualized severity levels. Early trajectories showed low, stable weekly PHQ-9 scores but greater GAD-7 variability**—**a pattern the patient reported aligned with his lived experience of fluctuating anxiety, reinforcing early confidence in visualization accuracy. In Week 2, when both scores spiked (red boxes in Figure 4), the upward trajectory was visible in the graphs. The DN helped him connect this change to close friends moving out of state. The subsequent decline (blue boxes in Figure 4) as he returned to structured coping strategies (completing 5 journal entries) was equally visible in the graphs’ return to baseline, reinforcing that his data accurately reflected his experiences.

Critically, comparing daily versus weekly symptom graphs revealed an important clinical distinction. Daily depression ratings showed high variability while weekly PHQ-9 scores remained stable. The DN used this visual contrast, variable daily graphs versus stable weekly graphs, to help the patient understand that daily ratings reflected mood-level fluctuations rather than clinical depressive symptoms. This visualization-anchored reframing prevented misinterpretation of normal emotional variability as symptom worsening.

### Distinguishing Situational From Persistent Patterns

**Figure 5.**
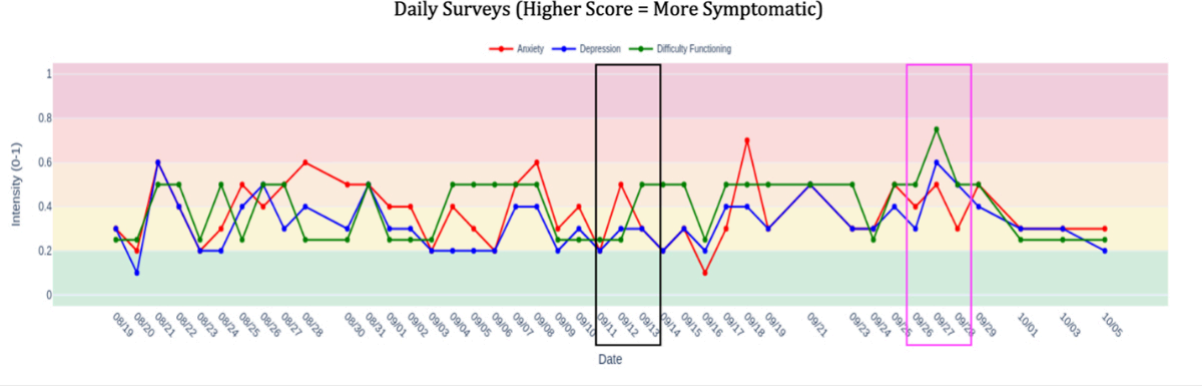
Daily symptom trajectory graph.

During illness and academic pressures, weekly GAD-7 scores showed a slight but steady decrease in the line graph, while daily anxiety and difficulty functioning temporarily spiked around Week 3 (black box in Figure 5). The contrast between stable weekly trend lines and volatile daily graphs enabled the DN to help the patient distinguish expected situational fluctuations from persistent symptom patterns. This visual evidence, that underlying improvement continued despite temporary setbacks, maintained therapeutic momentum and prevented discouragement during a challenging period.

### Independent Pattern Recognition

**Figure 6.**
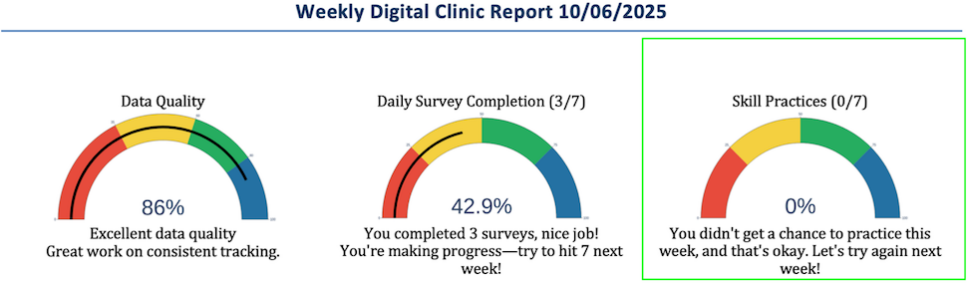
Week 6 gauge graphs.

By Week 6, the patient independently identified a brief symptom spike (pink box in Figure 5) in the line graphs without the DN prompting, demonstrating autonomous data interpretation skills. More significantly, symptom trajectory graphs showed continued decline in weekly anxiety even as skills practice engagement dropped to 0% (green box in Figure 6). This visual dissociation between declining symptom severity and declining formal engagement provided evidence that the patient had successfully internalized coping strategies, with the graphs anchoring validation of his growing autonomy in symptom management.

### Identifying Behavioral Targets and Verifying Predictions

Heat map visualizations displaying Spearman correlation coefficients between passive behavioral metrics (GPS-derived entropy, step count, hometime, and screen time) and active self-report measures (daily anxiety, depression, and difficulty functioning) served as anchors for exploring and validating behavioral-symptom relationships. The color-coding system: warm colors indicating positive correlations, cool colors showing negative correlations, with intensity reflecting strength, made complex statistical relationships easily interpretable.

### Learning to Read Behavioral-Symptom Relationships

**Figure 7.**
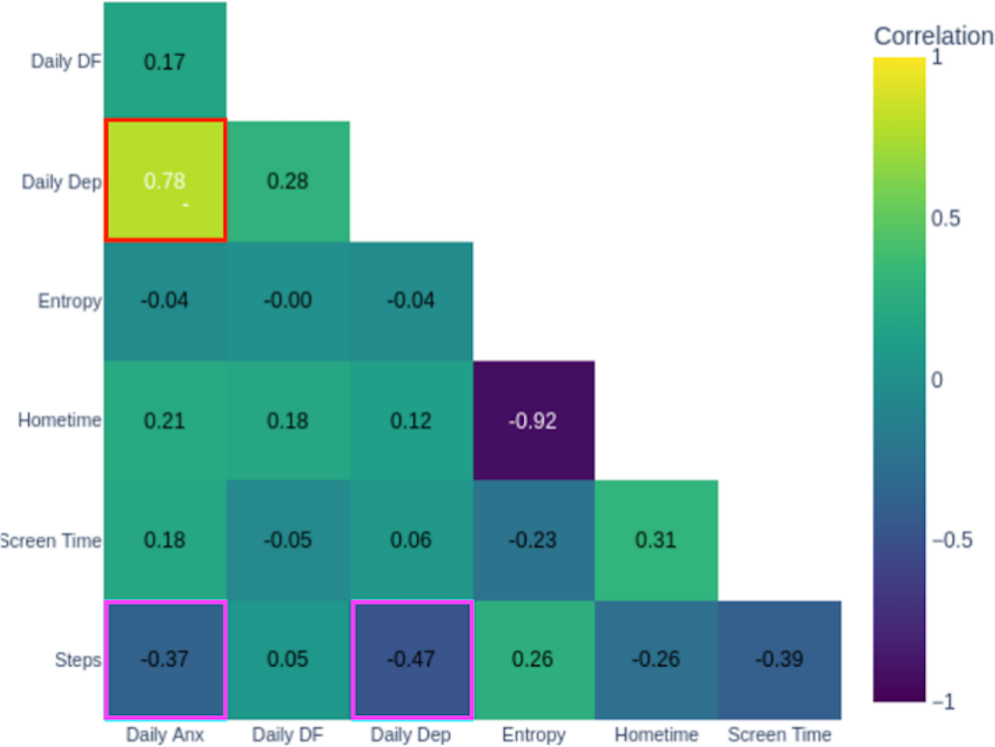
Week 2 correlation matrix. DF: daily functioning.

**Figure 8.**
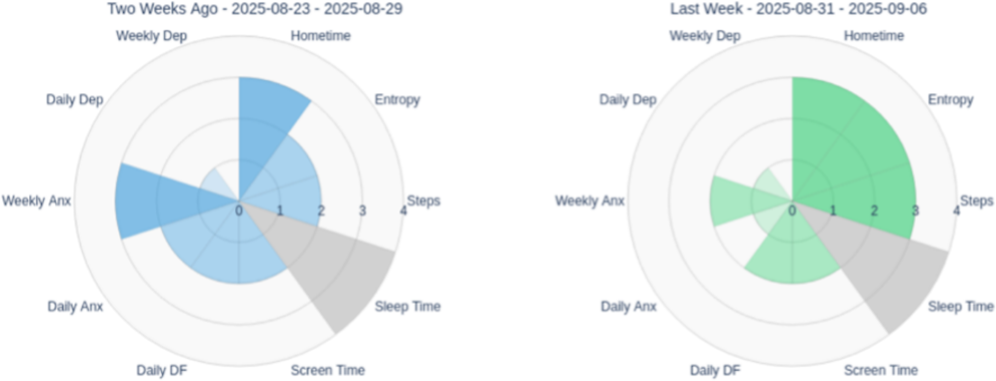
Week 2 passive-active data integration graphs. DF: daily functioning.

The DN introduced the patient to the correlation matrix by explaining how to interpret the heat map colors and what each axis represented. The matrix revealed that daily anxiety and daily depression showed positive correlations (red box in Figure 7), aligning with the patient’s lived experience that anxiety episodes worsened his mood. More actionably, the matrix showed negative correlations (pink boxes in Figure 7) between step count and daily anxiety and depression, indicating that lower mobility may correspond with heightened anxiety. The DN emphasized that weekly and daily anxiety decreased when entropy and step count increased—a pattern visible in Figure 8. The patient validated this against his experience, noting that he felt worse on days he stayed indoors or skipped exercise. This validation, anchored in the visual representation, transformed an abstract statistical relationship into a personally meaningful and actionable insight: behavioral activation through movement and routine variety could potentially reduce his anxiety.

### Contextualizing Disruptions Through Behavioral Data

**Figure 9.**
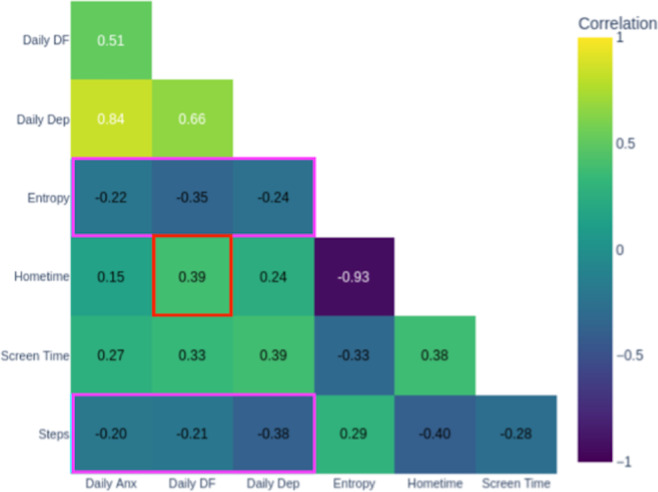
Week 4 correlation matrix. DF: daily functioning.

During illness in Week 4, the correlation matrix became an anchor for understanding why symptoms temporarily worsened. The matrix showed that hometime was positively correlated with difficulty functioning (red box in Figure 9), reflecting days spent recovering at home, while entropy and steps remained negatively correlated with daily anxiety, depression, and difficulty functioning (pink boxes in Figure 9), consistent with disrupted routines. The DN used these visual patterns to help the patient understand that his symptoms increased during illness were contextually appropriate and linked to necessary recovery behaviors rather than indicating treatment failure. The patient also noted anticipating future increases in difficulty functioning with increased screen time as classes intensified—demonstrating he was using the correlation matrix to make predictions about future patterns. The matrix thus anchors not only behavioral target identification but also appropriate contextualization of symptom changes within life circumstances.

### Data Integration and Autonomous Patient Predictions

**Figure 10.**
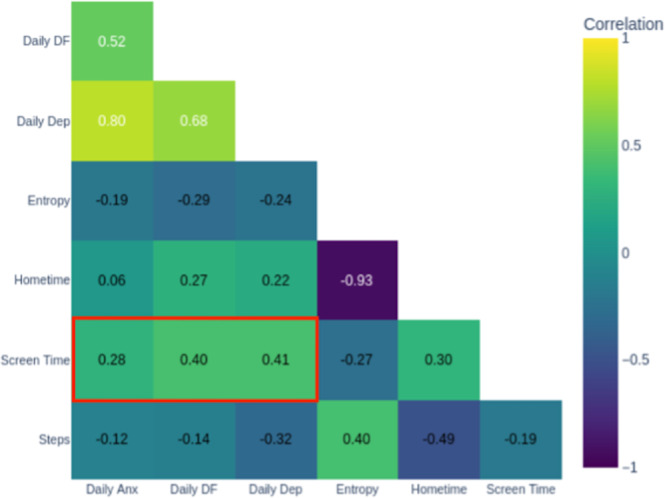
Week 6 correlation matrix. DF: daily functioning.

**Figure 11.**
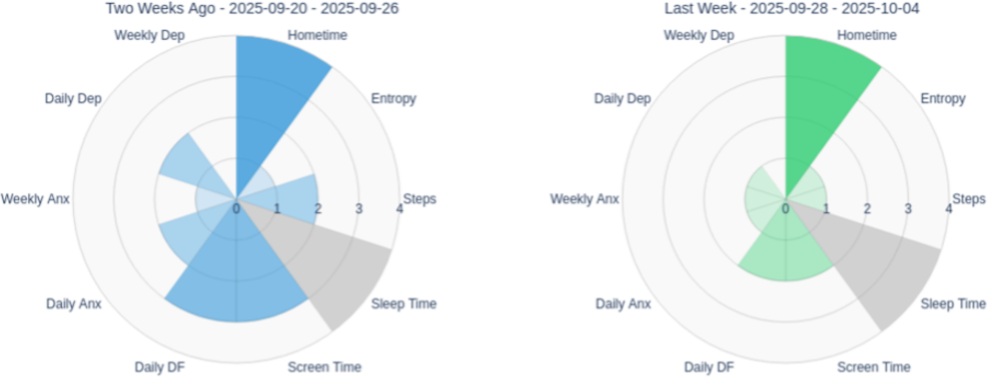
Week 6 passive-active data integration graph. DF: daily functioning.

By Week 6, the patient autonomously used the correlation matrix to generate and test assumptions made from the previous weeks. He noted that screen time’s positive correlation with both daily difficulty functioning and depression (red box in Figure 10) aligned with his academic workload and prior discussions about cognitive strain. Observing that both screen time and symptom scores had decreased that week (Figure 11), he independently anticipated that reduced screen time contributed most to his decreased scores. This analysis, integrating information from the correlation matrix, symptom trajectory graphs, and behavioral tracking data, demonstrated the ability to use visualizations as cognitive tools for understanding his mental health.

### Recognizing Multidimensional Change and Validating Progress

Radar charts displaying scores across multiple domains, such as depression, anxiety, difficulty functioning, and positive psychological metrics (emotional self-awareness, perceived social support, self-efficacy, motivation, digital literacy), at 3 time points (intake-red, interim-blue, and completion-green) served as pillars for recognizing comprehensive treatment effects beyond single symptom reduction. The radar format’s visual structure made multidimensional comparisons easily comprehensible: outward expansion indicated improvement in positive domains, while inward reduction showed reduction in symptom severity and functional impairment.

### Establishing Baseline and Visual Data Literacy

**Figure 12.**
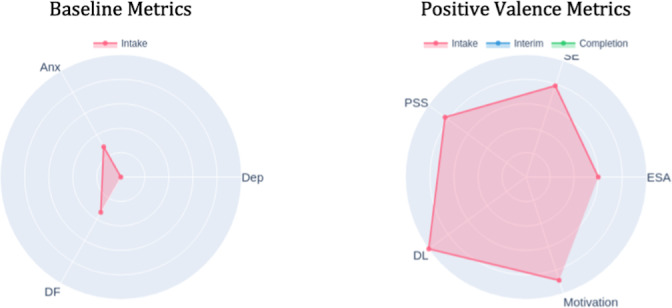
Intake polar chart. DF: daily functioning; DL: digital literacy; ESA: emotional self-awareness; PSS: Perceived Stress Scale.

At intake, the DN introduced the polar chart format by explaining how to read the radar pattern. The pink triangle showed higher anxiety and difficulty functioning relative to depression through the distinctive shape, with anxiety and difficulty functioning axes extended further outward, visually emphasizing anxiety as the primary concern (Figure 12). The chart also showed consistently elevated positive-valence measures, visible as outward extension on those axes, suggesting existing strengths. This visual representation validated the patient’s subjective experience and helped establish shared understanding between the patient, DN, and clinician about treatment priorities. The DN explained that future reports would overlay interim and completion patterns to show change over time.

### Visualizing Progress Despite Challenges

**Figure 13.**
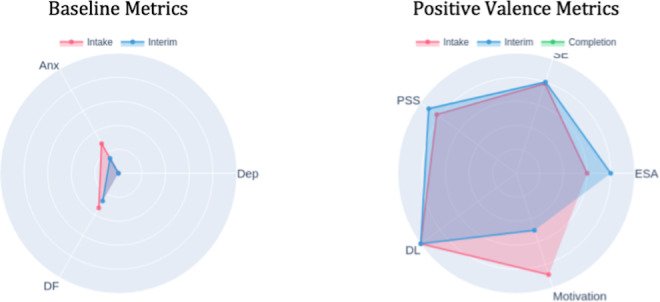
Interim polar chart. DF: daily functioning; DL: digital literacy; ESA: emotional self-awareness; PSS: Perceived Stress Scale.

The interim polar chart (blue) overlaid on the intake measures (pink) made improvements immediately apparent despite ongoing illness and academic pressures (Figure 13). The blue line pulled inward on the anxiety and difficulty functioning axes while extending outward on emotional self-awareness and perceived social support axes. The DN used this visual comparison to help the patient connect these multidimensional gains to his growing use of coping strategies, including meditation practices he had begun incorporating. The radar format made these changes more noticeable than reviewing individual scale scores, as the patient could see his overall psychological profile shifting toward improved mental health. This visual validation was particularly significant during a challenging week; the polar chart helped anchor his sense of meaningful progress despite ongoing challenges and reinforced that continued engagement itself was an achievement.

### Validating Comprehensive Growth

**Figure 14.**
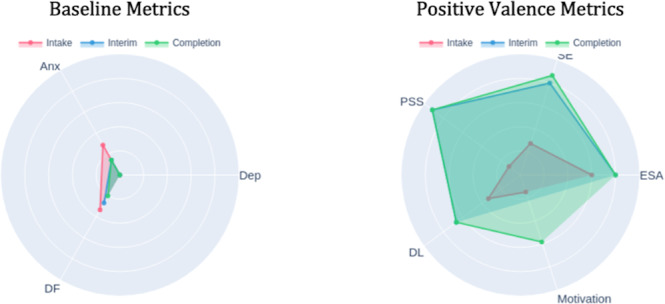
Completion polar chart. DF: daily functioning; DL: digital literacy; ESA: emotional self-awareness; PSS: Perceived Stress Scale.

At completion, the green radar pattern showed sustained improvements across domains: difficulty functioning had contracted further inward compared with both intake and interim, indicating functional gains; anxiety and depression remained stable at reduced levels; and positive metrics, particularly motivation and self-efficacy, had expanded outward beyond interim levels (Figure 14). The visualization displays overlapping time points—intake (pink), interim (blue), and completion (green)—with darker shaded regions indicating where multiple assessments overlap. The DN used this visualization to anchor the final session’s review of overall progress. The patient could see that his emerging sense of control and confidence in his subjective experience was reflected objectively in the change patterns. The polar chart format made the comprehensiveness of his improvement immediately visible: not just symptom reduction but psychological growth across multiple domains. This visual validation supported his confidence in maintaining gains posttreatment and reinforced that the self-monitoring skills he had developed were grounded in objective evidence of growth. The contrast between low formal skills practice completion (0% in wk 6) and continued psychological improvement validated that he had successfully internalized coping strategies.

The progression across these 3 visualization types illustrates how the DN supports development from guided interpretation to autonomous engagement. Each phase built upon the previous. In the guide phase, the DN taught how to read each visualization format and helped validate patterns against lived experience. In the refinement phase, visualizations anchored understanding of how symptoms related to life circumstances and behavioral patterns, supporting adaptive treatment planning during challenges. In the autonomy phase, the patient independently used multiple visualization types altogether, integrating information from trajectories, correlation matrices, and polar charts to generate predictions and validate his subjective sense of improvement through objective data patterns.

This case demonstrates how thoughtfully designed, multi-format data visualizations, when delivered through structured DN facilitation, can transform passive data collection into active clinical tools. The visualizations enhanced patient engagement, promoted behavioral insight, supported symptom improvement, and ultimately facilitated autonomous self-management—outcomes that extended beyond what traditional clinical monitoring or standalone digital tools typically achieve in digital mental health care.

## Discussion

### Principal Results

This paper presents a model for integrating data visualizations into digital mental health care through a DN-supported workflow. Our case demonstrates that when complex digital phenotyping data is translated into interpretable visual formats and delivered through structured support, it can transform passive data collection into active insight-building that enhances patient engagement, contextualizes symptom fluctuations, and supports real-time treatment adaptation.

This case highlights a key advantage of integrating data visualizations into routine digital mental health workflows: the patient remained engaged with visual reports even when his use of app-based exercises declined to 0% in his final week. Interestingly, this pattern suggests that visualizations may serve as an anchor for maintaining therapeutic momentum during periods of lower formal engagement with other digital intervention components. Such a finding has important implications for retention and sustained participation in digital interventions, where fluctuations in engagement represent a common concern. Despite reduced active skill practice, the patient’s continued interest in reviewing his weekly reports indicates that well-designed visualizations can sustain connection to treatment. A tiered approach offering progressive levels of detail may enhance accessibility for patients with varying preferences and literacy needs, while allowing more engaged individuals to explore deeper inferences from their data.

Second, the alignment between clinical trends and visualized data supports the feasibility of using these tools to track progress and guide treatment planning in real time. Clinician care coordination was enhanced through the DN’s ability to relay data-informed insights, ensuring that behavioral patterns identified in weekly reports expanded therapeutic discussions during clinician sessions. The DN role is critical to this coordination: by serving as an interpreter and facilitator of the visualized data, the DN enabled efficient information transfer to the clinician without imposing a substantial burden. This finding suggests that with an organized workflow design, data visualizations can meaningfully augment traditional therapy by making passive monitoring data clinically actionable.

Additionally, across the treatment period, the review of the data visualizations helped transform raw data into actionable clinical insight by connecting concrete life events, such as illness, academic demands, and social changes, to emerging symptom patterns. This contextualization process proved essential for meaningful interpretation. For example, the patient’s ability to link increased anxiety scores to specific academic stressors, or to recognize the impact of social schedule changes on his mood patterns, demonstrated how visualizations can become therapeutically valuable when paired with reflective discussion. The DN facilitated this interpretive process by prompting consideration of environmental and behavioral context, helping the patient move from passive data consumption to active insight-building. This outcome underscores the importance of designing visualizations that support nuanced interpretations accounting for everyday stressors, rather than suggesting rigid behavioral prescriptions disconnected from lived experience.

This case also suggests important directions for future refinement of the DN’s role. The patient’s ability to generate insightful predictions about behavioral-symptom relationships raises questions about optimal timing and intensity of DN support. Future work should examine whether DN involvement can be gradually reduced as patients develop data literacy and interpretive competence, or whether ongoing facilitation remains necessary to maintain engagement and insight quality. Additionally, further research is needed to determine which patient characteristics predict successful transition to more independent data interpretation versus continued need for facilitated review.

Finally, as digital phenotyping becomes increasingly integrated into mental health treatment, scalable models for making this data actionable will be essential. The data visualizations represent a replicable model that balances automation (standardized data processing and visualization generation) with personalization (individualized interpretation and contextualization). This may be particularly valuable for health systems seeking to implement digital phenotyping without overwhelming clinicians or expecting patients to navigate their data independently. The findings support the scalability potential of visualization-enhanced digital care models when key design principles are met. The DN role enables patients to develop both immediate symptom management insights and longer-term self-monitoring skills, representing an investment in patient capacity-building that may yield benefits beyond the acute treatment period. Further research should examine the cost-effectiveness of this model across diverse clinical settings and patient populations, as well as strategies for training and supervising DNs to ensure consistent quality of data facilitation.

### Comparison With Prior Work

Our findings align with and extend prior research on data visualization in digital mental health. Previous work has demonstrated that clinicians are receptive to digital phenotyping data when interpretation support is available [11], and our structured DN workflow provides a practical model for delivering such support. The visualization types we developed address the need for actionable information summarized from raw behavioral data [13], while the participatory design process ensured alignment with clinical workflow feasibility.

Our approach differs from previous data visualization tools [8] by embedding visualizations within a structured human support model rather than providing standalone tools for independent patient use. ESMvis and similar clinical dashboards [9] are primarily designed for researchers or clinicians’ use without guided patient interpretation workflows. Our model introduces the DN as a bridge between data collection and meaningful understanding, reflecting evidence that patients willingly share data when they can derive insights with trusted support [14]. This design decision reflects evidence that patients express willingness to share personal data when they can derive meaningful insights with trusted support [14]. The DN model addresses many of the implementation barriers identified in prior digital phenotyping research by providing dedicated personnel for technical troubleshooting, data interpretation, and care coordination.

An important issue is how our approach relates to therapeutic alliance. Although we did not formally assess alliance with validated instruments, qualitative interviews at treatment end provided relevant insights: patients reported that reviewing data with the DN was “helpful,” that they “understood their data more,” and that the experience became “more meaningful.” These findings suggest that collaborative data interpretation may itself serve as a relational intervention involving trust, validation, and shared understanding. Future research should use validated alliance measures (eg, the Working Alliance Inventory) to formally examine this relationship.

### Implementation Considerations and Scalability

The DN role was developed to help ease clinician burden in the Digital Clinic, with several considerations relevant to broader implementation [22]. In terms of scalability, the DN model is designed to be adaptable across diverse clinical contexts. Because the role does not require clinical licensure, DNs can come from various professional backgrounds, depending on available resources and target populations. The structured training and supervision framework described in the Methods section provides a replicable model for onboarding DNs in new settings. However, feasibility depends on institutional capacity to support dedicated DN positions, and research on cost-effectiveness is needed to assess sustainability across different health systems.

Regarding data governance and privacy, the mindLAMP platform is open-source and designed with privacy protections in mind [26]. All passive data are aggregated and deidentified. Data collected through the Digital Clinic is managed under institutional data-use agreements and HIPAA-compliant protocols. During onboarding, patients are informed about what data is collected, how it is processed, and who can access the resulting reports. Currently, patients cannot directly download raw data from mindLAMP. However, they can request static copies of their visualization reports. When a patient expresses interest in retaining their reports for future reflection, their DN generates and emails a PDF containing their weekly summary reports, which provides access to their data even after the 8-week program has ended. Patients may also request these reports retrospectively, and DNs respond by sharing the relevant materials via secure email. The mindLAMP platform does not share individual patient data with third parties, and any future policy changes would require updated consent procedures.

For engagement monitoring and clinical safety, DNs track engagement metrics such as survey completion, app usage, and session attendance. When engagement or data quality declines, the DN addresses potential barriers in the next session and adjusts the support plan as needed. If disengagement persists, the supervising clinician is alerted, and a coordinated care plan is established. Data reports highlight low data quality or completion, and clinicians receive alerts when symptom scores exceed clinical thresholds. At present, there is no formal protocol specifying DN involvement when a patient is hospitalized or for follow-up contact with families in the event of a patient’s death during the program. These scenarios are currently managed through standard clinical workflows outside the scope of the DN role, and the model does not systematically incorporate bereavement outreach or posthospitalization navigator support. Developing structured procedures for DN participation in these critical events represents an important area for future work, particularly given the potential role of DNs in providing support and continuity of care for patients and families during crises.

### Limitations

This study has several important limitations that should be considered when interpreting findings and planning future research. First, interpretation depends on patients’ subjective sense of whether visualizations align with their lived experience—a suitable approach given that mental health assessment already relies heavily on self-report measures like the PHQ-9 and GAD-7. However, patients may selectively validate patterns that confirm pre-existing beliefs while dismissing contradictory data. Future work should explore methods for balancing subjective validation with objective clinical markers. Second, these visualizations require smartphone ownership and willingness to share passive sensing data. While smartphone ownership is increasingly common, disparities remain across socioeconomic, age, and geographic groups. Data quality also varies between smartphone models, potentially introducing systematic bias [27,28]. The DN model addresses many of these concerns by helping patients troubleshoot technical issues and contextualize data quality indicators, though patients who are unwilling or unable to share data will be excluded from these approaches. Third, the case example shared to illustrate the potential of this approach may not generalize to other use cases, although in running this model of care for hundreds of patients, we have found the approach productive [29]. Finally, this study did not include formal measures of therapeutic alliance or patient satisfaction with the DN role, limiting our ability to make claims about the effects of this approach on the therapeutic relationship. Future studies should incorporate validated alliance measures to examine this dimension.

### Conclusions

Smartphones can generate unprecedented volumes of clinically relevant behavioral data, yet this data richness has limited clinical utility due to interpretation challenges. Our model demonstrates one pathway forward: joining thoughtfully designed visualizations with human support to bridge the gap between technical capability and clinical practice. Future research should examine whether the guide-refinement-autonomy progression generalizes across diverse populations, explore optimal models for training and supporting DNs, and investigate how visualization design can support nuanced interpretation that accounts for individual life circumstances. As the field moves toward increasingly data-driven care, models that center on human connection and collaborative interpretation will remain essential for ensuring that technology enhances rather than replaces the therapeutic relationship.

## Supplementary material

10.2196/90255 Multimedia Appendix 1 Technical development and data processing.

10.2196/90255 Multimedia Appendix 2 Python package table.

## Abbreviations

BIDMC: Beth Israel Deaconess Medical Center
DN: Digital Navigator
DOORS: Digital Outreach for Obtaining Resources and Skills
EMA: ecological momentary assessment
ESM: experience sampling method
GAD-7: Generalized Anxiety Disorder-7
PHQ-9: Patient Health Questionnaire-9

## References

[1] Cipriani A, Ward T, Lambe S (2025). Beyond counting clicks: rethinking engagement in digital mental health. Br J Psychiatry.

[2] Smith KA, Ward T, Lambe S (2025). Engagement and attrition in digital mental health: current challenges and potential solutions. NPJ Digit Med.

[3] Pew research center. Mobile fact sheet.

[4] Shanahan M, Bahia K (2025). The state of mobile internet connectivity 2025 – overview report. https://www.gsmaintelligence.com/research/research-file-download?reportId=66955&assetId=67095.

[5] Torous J, Linardon J, Goldberg SB (2025). The evolving field of digital mental health: current evidence and implementation issues for smartphone apps, generative artificial intelligence, and virtual reality. World Psychiatry.

[6] Linardon J, Chen K, Gajjar S (2025). Smartphone digital phenotyping in mental health disorders: a review of raw sensors utilized, machine learning processing pipelines, and derived behavioral features. Psychiatry Res.

[7] Barnett I, Torous J, Staples P, Keshavan M, Onnela JP (2018). Beyond smartphones and sensors: choosing appropriate statistical methods for the analysis of longitudinal data. J Am Med Inform Assoc.

[8] Bringmann LF, van der Veen DC, Wichers M, Riese H, Stulp G (2021). ESMvis: a tool for visualizing individual experience sampling method (ESM) data. Qual Life Res.

[9] Myin-Germeys I, Schick A, Ganslandt T (2024). The experience sampling methodology as a digital clinical tool for more person-centered mental health care: an implementation research agenda. Psychol Med.

[10] van Os J, Verhagen S, Marsman A (2017). The experience sampling method as an mHealth tool to support self-monitoring, self-insight, and personalized health care in clinical practice. Depress Anxiety.

[11] Schulte-Strathaus JCC, Ikegwuonu T, Schick A (2025). Visualization of experience sampling method data in mental health: qualitative study of the physicians’ perspective in Germany. J Med Internet Res.

[12] Rogan J, Firth J, Bucci S (2024). Healthcare professionals’ views on the use of passive sensing and machine learning approaches in secondary mental healthcare: a qualitative study. Health Expect.

[13] Sterling WA, Sobolev M, Van Meter A (2022). Digital technology in psychiatry: survey study of clinicians. JMIR Form Res.

[14] Torous J, Myrick K, Aguilera A (2023). The need for a new generation of digital mental health tools to support more accessible, effective and equitable care. World Psychiatry.

[15] Chang S, Gray L, Alon N, Torous J (2023). Patient and clinician experiences with sharing data visualizations integrated into mental health treatment. Soc Sci (Basel).

[16] Scheuer L, Torous J (2022). Usable data visualization for digital biomarkers: an analysis of usability, data sharing, and clinician contact. Digit Biomark.

[17] Chen K, Lane E, Burns J, Macrynikola N, Chang S, Torous J (2024). The digital navigator: standardizing human technology support in app-integrated clinical care. Telemed J E Health.

[18] Macrynikola N, Nguyen N, Lane E, Yen S, Torous J (2023). The digital clinic: an innovative mental health care delivery model utilizing hybrid synchronous and asynchronous treatment. NEJM Catalyst.

[19] Calvert E, Hackett K, Torous J, Giovannetti T (2025). Barriers and facilitators to usability of a smartphone-based digital mental health tool in older adults: insights from a secondary analysis of mindLAMP. Int Psychogeriatr.

[20] Macrynikola N, Chen K, Lane E (2025). Testing the feasibility, acceptability, and potential efficacy of an innovative digital mental health care delivery model designed to increase access to care: open trial of the digital clinic. JMIR Ment Health.

[21] Rodriguez-Villa E, Rauseo-Ricupero N, Camacho E, Wisniewski H, Keshavan M, Torous J (2020). The digital clinic: implementing technology and augmenting care for mental health. Gen Hosp Psychiatry.

[22] Wisniewski H, Torous J (2020). Digital navigators to implement smartphone and digital tools in care. Acta Psychiatr Scand.

[23] Burns J, Chen K, Flathers M (2024). Transforming digital phenotyping raw data into actionable biomarkers, quality metrics, and data visualizations using cortex software package: tutorial. J Med Internet Res.

[24] Byun AJS, Li Y, Cong S Sleep estimation from low frequency smartphone sensors via bayesian hidden markov model. In Review.

[25] Hoffman L, Wisniewski H, Hays R (2020). Digital opportunities for outcomes in recovery services (DOORS): a pragmatic hands-on group approach toward increasing digital health and smartphone competencies, autonomy, relatedness, and alliance for those with serious mental illness. J Psychiatr Pract.

[26] Torous J, Wisniewski H, Bird B (2019). Creating a digital health smartphone app and digital phenotyping platform for mental health and diverse healthcare needs: an interdisciplinary and collaborative approach. J Technol Behav Sci.

[27] Passell E, Strong RW, Rutter LA (2021). Cognitive test scores vary with choice of personal digital device. Behav Res Methods.

[28] Torous J, Staples P, Onnela JP (2015). Realizing the potential of mobile mental health: new methods for new data in psychiatry. Curr Psychiatry Rep.

[29] Calvert E, Cipriani M, Chen K, Dhima A, Burns J, Torous J (2025). Evaluating clinical outcomes for anxiety and depression: a real-world comparison of the digital clinic and primary care. J Affect Disord.

