# Supplementary material for: Integrating Data Visualizations Into Digital Mental Health Care for Adults With Anxiety and Depression: Participatory Design and Case Study
Source: J Particip Med. 2026 Apr 24;18:e90255. doi: 10.2196/90255 (PMC13108835; doi:10.2196/90255)
Supplement: Multimedia Appendix 1 [file jopm-v18-e90255-s001.docx]

**Appendix 1. Technical Development and Data Processing**

All data visualizations were generated using Python 3 in a Jupyter Notebook environment. Cortex, an open-source pipeline created by the Division of Digital Psychiatry for mindLAMP-based studies, retrieves raw ecological momentary phenotyping data from a central database. Cortex has been validated as a secure and standardized tool used by over 100 research teams worldwide [1].

mindLAMP collects self-reported data (e.g., weekly PHQ-9, GAD-7, daily mood ratings) and passive behavioral data via smartphone sensors, including GPS-derived hometime, accelerometer-based movement, and screen-state activity patterns. All passive signals are aggregated and de-identified; precise locations and raw GPS traces are never stored. Sleep metrics were derived using a Bayesian hidden Markov model integrating accelerometer and screen state data [2].

Python libraries used: Pandas and Numpy (data cleaning, manipulation, transformation), datetime (timestamp conversion), Scipy and Sklearn (statistical analysis), Seaborn, Matplotlib, and Plotly (visualizations), and Docx (report generation). Please see Multimedia Appendix 2 for detailed package lists and functions.

References:

1. Burns J, Chen K, Flathers M, et al. Transforming digital phenotyping raw data into actionable biomarkers, quality metrics, and data visualizations using cortex software package: tutorial. J Med Internet Res. Aug 23, 2024;26:e58502. [doi: 10.2196/58502] [Medline: 39178032]

2. Byun AJS, Li Y, Cong S, et al. Sleep estimation from low frequency smartphone sensors via bayesian hidden markov model. Research Square. Preprint posted online on Aug 6, 2025. [doi: 10.21203/rs.3.rs-7217304/v1]
