# Supplementary material for: Integrating Data Visualizations Into Digital Mental Health Care for Adults With Anxiety and Depression: Participatory Design and Case Study
Source: J Particip Med. 2026 Apr 24;18:e90255. doi: 10.2196/90255 (PMC13108835; doi:10.2196/90255)
Supplement: Multimedia Appendix 2 [file jopm-v18-e90255-s002.docx]

**Appendix 2. Python Package Table**

| **Package Names** | **Version** |
| --- | --- |
| Altair | 4.2.0 |
| Calplot | 0.1.7.5 |
| Cortex | N/A |
| LAMP | N/A |
| Matplotlib | 3.4.1 |
| Numpy | 1.22.3 |
| Pandas | 1.4.2 |
| Plotly | 5.15.0 |
| Seaborn | 0.11.1 |
| SciPy | 1.10.1 |
| Python-docx | 1.1.2 |
| Sklearn | 0.0 |
| Scikit-learn | 0.24.1 |
| Scikit-image | 0.18.1 |
